# Machine learning modeling of vegetation and limited two dimensional urban morphology effects on land surface temperature in Osaka using open data

**DOI:** 10.1038/s41598-026-49813-4

**Published:** 2026-05-21

**Authors:** Xiong Xiao, Yasuhiro Shimazaki, Marko Bizjak, Zhichao Jiao, Jiale Chai, Xiangfei Kong, Yafeng Gao, Gangyi Tan, Yan Ding, Zhe Tian, Da Yan, Jihui Yuan

**Affiliations:** 1 Dept. of Living Environment Design, Grad. School of Human Life and Ecology, Osaka Metropolitan University, Osaka, 5588585 Japan; 2 Dept. of Architecture and Civil Eng., Grad. School of Eng, Toyohashi University of Technology, Aichi, Japan; 3 Faculty of Electrical Engineering and Computer Science, University of Maribor, Koroška cesta 46, Maribor, 2000 SI Slovenia; 4 School of Architecture, Yantai University, Yantai, 264005 China; 5 School of Energy and Environmental Engineering, Hebei University of Technology, Tianjin, 300401 PR China; 6 Joint International Research Laboratory of Green Building and Built Environment, Ministry of Education, Chongqing University, Chongqing, 400044 China; 7 School of Architecture and Urban Planning, Huazhong University of Science and Technology, Wuhan, 430074 Hubei PR China; 8 School of Environmental Science and Engineering, Tianjin University, Tianjin, 300072 PR China; 9 Building Energy Research Center, School of Architecture, Tsinghua University, Beijing, PR China

**Keywords:** Urban heat island, Land surface temperature, Urban morphology, Vegetation indices, Machine learning, Climate sciences, Ecology, Ecology, Environmental sciences

## Abstract

Urban heat islands (UHI) significantly elevate land surface temperatures (LST) in high-density subtropical cities like Osaka, Japan, exacerbating energy demand, health risks, and climate vulnerability. This study investigates LST drivers using freely accessible Landsat 9 data (August 27, 2024) and OpenStreetMap (OSM) building footprints at 100 m resolution. Due to the absence of reliable 3D height data, we focus on vegetation indicators (mean NDVI and vegetation fraction) and basic 2D morphology (building coverage ratio [BCR] and building area density ratio [BADR], assuming uniform 10 m height) as a pragmatic open-data baseline. Vegetation metrics show weak positive associations with LST (Pearson *r* = 0.173 for NDVI_mean, 0.114 for VegFrac), while 2D morphology exhibits negligible links (*r* ≈ 0.008). These results highlight the limited explanatory power of planimetric indicators alone in humid subtropical settings. Machine learning models (Random Forest [RF], XGBoost [XGB], Artificial Neural Network [ANN]) substantially outperformed multiple linear regression (R² = 0.045), with XGB achieving the highest performance (R² = 0.233, RMSE = 29.6 °C). NDVI_mean dominated feature importance (55.3%). Spatial predictions identified LST hotspots in central districts (high BCR, low vegetation), where partial dependence analysis suggests an indicative marginal LST reduction of ≈ 1.0–1.5 °C associated with a 10% point increase in VegFrac (model-derived statistical association, not causal; subject to considerable uncertainty due to the modest R² and excluded confounders). Results emphasize the need for multi-variable frameworks incorporating 3D morphology (e.g., sky view factor, height variation), landscape patterns, and meteorological factors to enhance predictive accuracy and inform targeted greening, ventilation corridors, and cool materials in Osaka’s urban planning. As a replicable, low-cost open-data baseline, this study offers practical insights for resource-constrained subtropical cities, contributing to Sustainable Development Goals (SDGs) 11 and 13.

**Supplementary Information:**

The online version contains supplementary material available at 10.1038/s41598-026-49813-4.

##  Introduction

### Background on urban heat islands and urban morphology

The urban heat island (UHI) effect, defined as the elevated temperature of urban areas relative to surrounding rural zones, arises primarily from anthropogenic modifications of land surfaces and energy use^1,2^. The phenomenon primarily arises from the replacement of natural surfaces with impervious materials such as concrete and asphalt, which reduce evapotranspiration and increase heat storage within the built environment. Consequently, heat accumulates and radiates within urban canyons, intensifying nighttime temperatures and increasing the demand for cooling energy^3,4^.

UHI intensification leads to multiple socio-environmental challenges, including elevated energy consumption, air quality deterioration, and adverse health impacts during extreme heat events^5–7^. Even small-scale urban modifications, such as elevator additions to buildings, can alter community microclimates by affecting airflow and shading, contributing to UHI intensification^8^. In humid subtropical cities such as Osaka, Japan, the effect is exacerbated by high humidity, limited ventilation, and persistent summer heatwaves^9^. Observational and modeling studies have reported temperature differentials exceeding 10 °C between Osaka’s dense urban core and its peripheral green spaces^10,11^, highlighting the need for a detailed understanding of local UHI mechanisms.

Urban morphology—defined by the density, geometry, and spatial configuration of buildings—plays a crucial role in regulating surface energy balance and heat distribution^12–14^. Metrics such as Building Coverage Ratio (BCR) and Building Area Density Ratio (BADR) are positively correlated with land surface temperature (LST) due to reduced air circulation and increased thermal mass^15,16^. Conversely, vegetation-related indicators, such as the Normalized Difference Vegetation Index (NDVI) and Vegetation Fraction (VegFrac), generally show negative correlations with LST because vegetation enhances evaporative cooling and provides shading^17–20^. However, in compact high-density environments, this cooling relationship often becomes nonlinear or even reverses due to heat stress on vegetation and reduced shading efficiency^21,22^.

Given Osaka’s diverse urban landscape—ranging from commercial high-rises to suburban parks—the city offers an ideal case to examine how urban form and vegetation jointly influence surface temperature. Such understanding is essential for informing climate-resilient urban design and supporting Sustainable Development Goals related to energy efficiency and livability.

### Machine learning applications in UHI studies

Machine learning (ML) techniques have recently become essential tools in urban climate research, offering improved capacity to capture complex, nonlinear interactions between LST, vegetation, and morphology compared with conventional regression models^23–25^. Traditional linear methods often fail to represent the multiscale dependencies and feedbacks inherent in urban systems, resulting in reduced predictive accuracy^12,26^.

Among the ML algorithms, Random Forest (RF), Extreme Gradient Boosting (XGB), and Artificial Neural Networks (ANN) are widely used for UHI prediction^24,27^. RF provides robustness and interpretability through variable importance measures, XGB achieves high efficiency and generalization, and ANN captures continuous nonlinear relationships typical of complex thermal processes^28–30^. For instance, RF-based analyses in Chinese megacities have effectively linked 3D building form and vegetation configuration to heat distribution patterns^13,15^, while XGB and ensemble models have demonstrated strong potential in assessing climate adaptation strategies across different climatic contexts^31,32^.

However, model performance depends heavily on the comprehensiveness of predictor variables. Studies incorporating topographic, meteorological, and land-use features consistently report higher predictive power (R² > 0.8) than those relying solely on vegetation indices or two-dimensional morphology^25^. Thus, integrating multi-source datasets—including 3D urban morphology and satellite-derived vegetation metrics—remains key to advancing UHI modeling accuracy and interpretability.

### Research gaps and objectives

Despite significant advancements in ML-based UHI studies, notable research gaps persist in humid subtropical Asian contexts, where high humidity, dense morphology, and seasonal heat stress mediate complex thermal interactions between built-up and vegetated surfaces. Many prior studies have examined vegetation indices or morphological parameters in isolation, often overlooking their combined and potentially interactive effects on LST^33,34^. In Japan, relatively few investigations have applied high-resolution spatial data and ML techniques to quantify these relationships at the city scale^14,15^.

Recent research has increasingly incorporated a broader suite of urban morphology indicators—including mean building height, floor area ratio (FAR), sky view factor (SVF), frontal area index, road density, and landscape pattern metrics (e.g., patch density, edge density, shape complexity)— to better capture three-dimensional (3D) and configurational effects on LST (e.g.,^14,35^; see also studies comparing plain vs. plateau cities and seasonal/diurnal variations). Such multi-metric approaches typically achieve higher explanatory power. However, reliable 3D building height data are frequently unavailable or inconsistent in open-access sources such as OpenStreetMap (OSM) for large Japanese cities like Osaka. Consequently, many resource-constrained analyses — particularly those relying solely on freely available OSM building footprints — are limited to basic planimetric (2D) density metrics such as BCR and BADR.

This study deliberately adopts such a constrained approach as a pragmatic starting point. Recent studies have demonstrated the superior explanatory power of 3D morphology metrics (e.g., building height, sky view factor, frontal area index) over 2D indicators in capturing seasonal and diurnal LST variations, particularly in summer when vertical structure strongly influences heat trapping and ventilation^36–38^. By focusing on BCR and BADR (with BADR estimated under a uniform 10 m height assumption due to the absence of height information), we explicitly test whether simple 2D morphological indicators provide meaningful additional explanatory value beyond vegetation metrics in a humid subtropical high-density setting where reliable 3D data are often unavailable or inconsistent in open sources like OSM. This addresses a key gap: while advanced 3D/multi-metric approaches achieve high R² (> 0.7), few studies provide replicable baselines using only freely accessible 2D data for resource-constrained subtropical Asian cities, where vegetation cooling is frequently limited by humidity and mixed land cover.

To address the identified gaps, this study:

(i) Computes and maps vegetation indicators (mean NDVI, vegetation fraction) and basic 2D morphology indicators (BCR, BADR) at 100 m resolution using Landsat 9 and OSM data;

(ii) Quantifies their spatial and statistical relationships with LST, including segmented and contextual analyses to better understand nonlinear patterns;

(ii) Develops and compares multiple ML models (Random Forest, XGBoost, Artificial Neural Network) for LST prediction, initially incorporating morphology but ultimately relying primarily on vegetation due to the weak performance of 2D metrics;

(iv) Interprets the findings in terms of urban heat mitigation priorities and climate-adaptive planning under open-data constraints.

The indicators (NDVI_mean, VegFrac, BCR, BADR) were selected because they are directly derivable from open-access Landsat 9 and OSM data, represent core biophysical (vegetation) and planimetric (density) drivers of LST widely used in prior UHI research, and allow a pragmatic baseline test of their combined explanatory power under severe 3D data limitations.

By integrating vegetation and basic morphological parameters within a unified ML framework — while transparently reporting the limited role of planimetric density — this study provides a replicable, low-cost baseline analysis for humid subtropical cities where advanced 3D datasets are often inaccessible. The work aims to improve understanding of UHI drivers under realistic data limitations and offer quantitative guidance for targeted greening and ventilation strategies that support sustainable urban development in resource-constrained environments.

## Materials and methodology

### Research framework

**Fig. 1:**
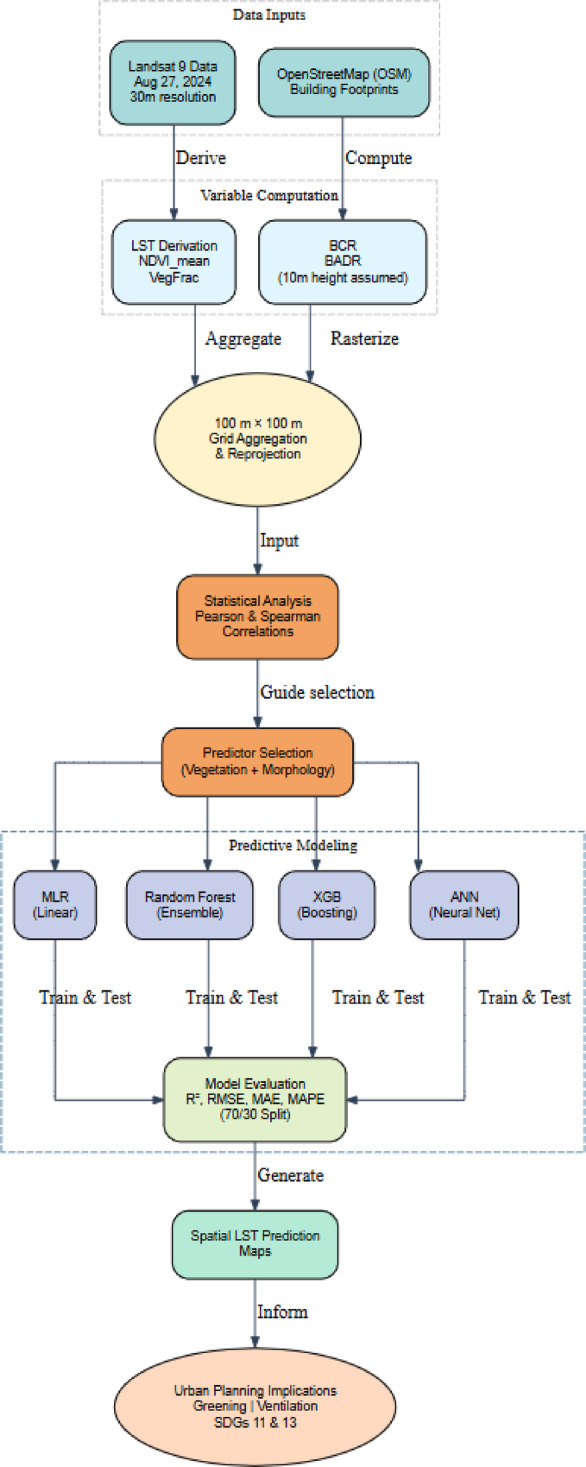
Research framework of this study.

The overall research framework of this study is illustrated in Fig. 1. The workflow integrates multi-source geospatial data, statistical analysis, and ML modeling to quantify the influence of urban morphology and vegetation on LST in Osaka (Kansei region), Japan.

The process begins with the acquisition of Landsat 9 satellite imagery (August 27, 2024) and OSM building footprints. From the Landsat data, LST, NDVI_mean, and VegFrac are derived at 30 m resolution. OSM building data are used to compute BCR and BADR, assuming a uniform building height of 10 m due to the absence oflogos of 3D height data. All variables are aggregated to a consistent 100 m × 100 m grid using bilinear interpolation and area-weighted averaging to ensure spatial alignment. BCR and BADR were selected as the primary urban morphology indicators because they are directly derivable from 2D OSM building footprints without requiring 3D height information, which is often unavailable or inconsistent in open datasets for large-scale Japanese cities. This choice aligns with many Landsat/OSM-based UHI studies focusing on planimetric density, although it omits vertical and configurational aspects (e.g., height variation, SVF, road network density) that could enhance explanatory power.

Statistical relationships between LST and the four urban indicators are assessed using Pearson and Spearman correlation coefficients. These correlations guide predictor selection for modeling.

Four predictive models are developed: Multiple Linear Regression (MLR) model, and three ML models (RF, XGB, and ANN). Models are trained on 70% of the data and evaluated on a 30% hold-out test set using performance metrics including coefficient of determination (R²), root mean square error (RMSE), mean absolute error (MAE), and mean absolute percentage error (MAPE).

The final outputs include spatial LST prediction maps, model performance comparisons, and urban planning recommendations targeting high-LST zones through enhanced greening and ventilation strategies, aligning with SDGs 11 (Sustainable Cities and Communities) and 13 (Climate Action).

### Study area

**Fig. 2:**
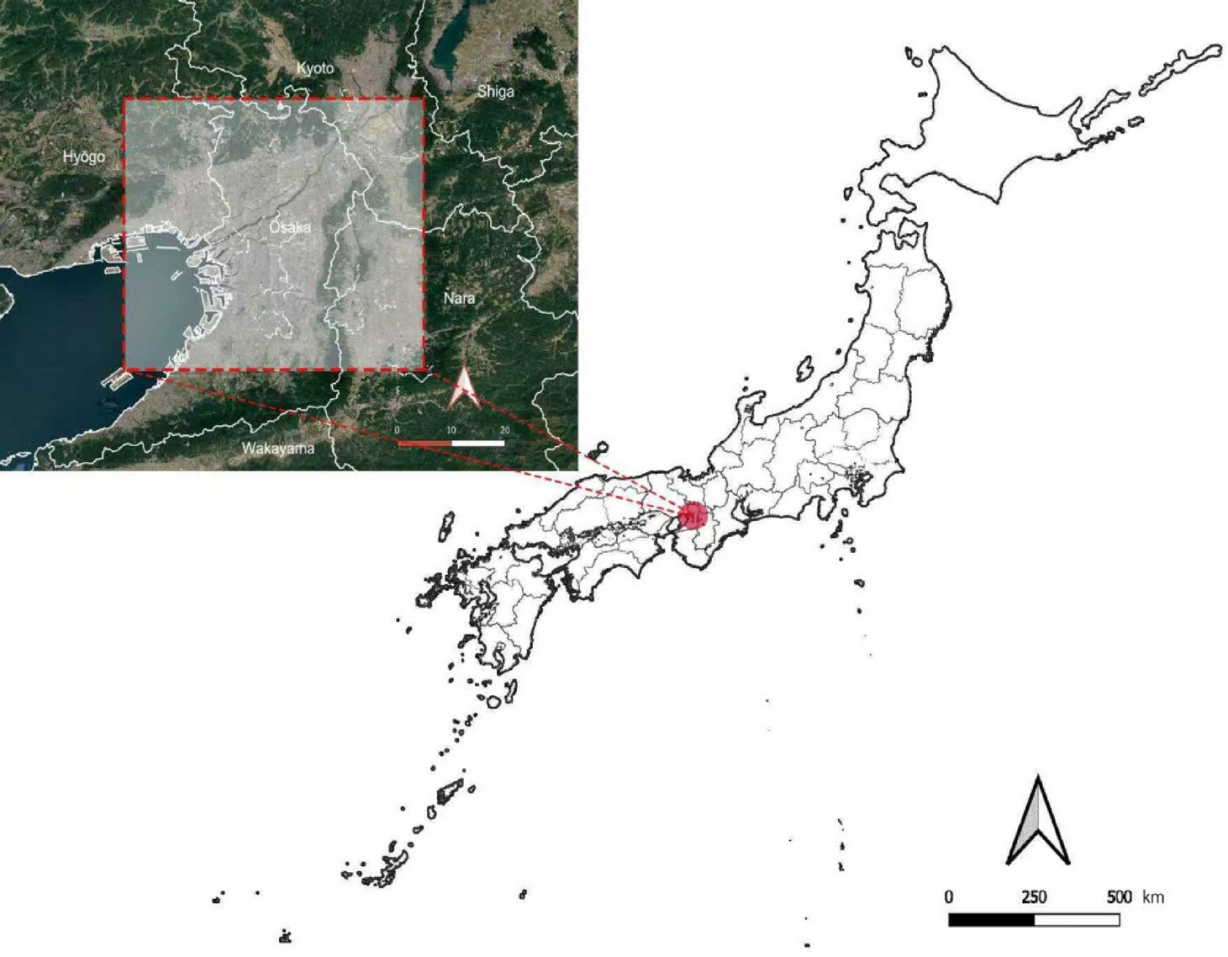
shows the geographic extent of the study area within Osaka, Japan.

The study area, Osaka (34.5°–34.9° N, 135.3°–135.8° E), covers approximately 223 km² in Japan’s Kansai region (Fig. 2). The city experiences a humid subtropical climate, characterized by hot, humid summers and mild winters (mean August temperature: 29.1 °C). Urban land use is dominated by high-density residential, industrial, and commercial areas, with relatively limited green spaces. Central districts, such as Umeda and Namba, exhibit a BCR greater than 0.5, whereas suburban parks, including Osaka Castle Park and Tennoji Park, show VegFrac exceeding 0.7, contributing to local cooling effects.

Figure 2. Geographic extent of the study area within Osaka, Japan. The map was created by the authors using QGIS (v3.34) based o on the administrative boundary data (N03-23_230101.shp downloaded from https://nlftp.mlit.go.jp/ksj/gml/datalist/KsjTmplt-N03-2024.html). The red dashed rectangle indicates the boundary of the study area used in the analysis (34.5°–34.9°N, 135.3°–135.8°E). Background shows the Kansai region for spatial context.

### Data sources

This study utilized Landsat 9 Collection 2 Level-2 data (Path/Row 110/036, August 27, 2024) for LST and NDVI derivation, combined with OSM building footprints for morphological variables (Table 1). Cloud cover was below 10%, ensuring high data quality.

**Table 1:** Data sources and specifications.

Data	Source	Date	Resolution
Landsat 9 OLI/TIRS Level-2	USGS EarthExplorer	27 Aug 2024	30 m (OLI), 100 m (TIRS)
Building Footprints	OpenStreetMap (gis_osm_buildings_a_free_1.shp)	2024	Vector

Note: *the data sources and access information are as follows*:.

- *Landsat 9 Collection 2 Level-2 (surface reflectance & surface temperature): USGS EarthExplorer —* https://earthexplorer.usgs.gov/*(Landsat → Landsat Collection 2 Level-2 → Landsat 8–9 OLI/TIRS C2 L2)*.
- *Building footprints: OpenStreetMap —* https://www.openstreetmap.org; *regional extract via Geofabrik —* https://download.geofabrik.de/asia/japan.html.

### Variable computation

All computations were performed in R (v4.3) using the packages “terra”, “sf”, “dplyr”, and “ggplot2”. All rasters were reprojected and aggregated to a 100 m grid to ensure consistency between satellite and vector datasets.

#### Land surface temperature (LST)

The Landsat Level-2 thermal band (Band 10, ST_B10) provides pixel-based radiance converted to at-sensor temperature (Kelvin) using scale and offset factors.

The raw thermal values were converted to degrees Celsius as follows:

1 $$\:LST(^\circ\:C)=(DN\times\:0.00341802+149.0)-273.15$$

where, $\:DN$ is the digital number from the ST_B10 band, 0.00341802 and 149.0 are the radiometric rescaling factors from the metadata (GAIN and OFFSET), subtraction of 273.15 converts Kelvin to Celsius.

The temperature raster was re-projected and aggregated to a 100 m grid using bilinear interpolation to match the urban morphology indices.

#### Normalized difference vegetation index (NDVI)

NDVI was derived from the Landsat surface reflectance bands:

2 $$\:NDVI=\frac{NIR-RED}{NIR+RED}$$

where $\:NIR$ is the reflectance of Band 5 (0.85–0.88 μm), $\:RED$ is the reflectance of Band 4 (0.64–0.67 μm). Pixels with $\:NDVI<-1$ or $\:NDVI>1$ were masked as invalid. The per-cell mean NDVI (NDVI_mean) was computed by averaging all valid NDVI values within each 100 m grid cell.

#### Vegetation fraction (VegFrac)

Vegetation fraction represents the proportion of vegetated pixels within each 100 m grid cell. It was estimated using a threshold of $\:NDVI>0.2$ (a common empirical indicator separating vegetated from non-vegetated surfaces):

3 $$\:VegFrac=\frac{{N}_{NDVI>0.2}}{{N}_{total}}$$

where $\:{N}_{NDVI>0.2}$ = number of pixels with NDVI > 0.2 inside the grid cell, $\:{N}_{total}$ = total number of valid pixels in that cell.

#### Building coverage ratio (BCR)

BCR quantifies the proportion of ground surface occupied by building footprints:

4 $$\:BCR=\frac{{A}_{bld}}{{A}_{cell}}$$

where $\:{A}_{bld}$ = summed planimetric area of building polygons within the grid cell (m²), $\:{A}_{cell}=100{\hspace{0.17em}}m\times\:100{\hspace{0.17em}}m=\mathrm{10,000}{\hspace{0.17em}}{m}^{2}$.

All building polygons were rasterized to the same 100 m grid using their footprint area attributes.

#### Building area density ratio (BADR)

BADR expresses the volumetric density of buildings relative to the cell area:

5 $$\:BADR=\frac{{V}_{bld}}{{A}_{cell}}$$

6 $$\:{V}_{bld}={A}_{bld}\times\:{H}_{bld}$$

where $\:{H}_{bld}$ = building height (m). In the absence of height data, a default mean height of 10 m was assumed. BADR thus approximates the built-up mass (m³/m²) and is correlated with both urban density and thermal storage capacity.

The selection of BCR and BADR was motivated by their ability to represent horizontal building density and approximate volumetric effects using a uniform height assumption. However, this limits the representation of full 3D urban form (e.g., height heterogeneity, sky view factor, or frontal area index), which recent studies have shown to be more influential in explaining LST variability (e.g.,^14^; see also analyses incorporating road density and landscape pattern indices).

BADR was included to test whether a simple volumetric proxy (building volume per unit ground area) could capture additional heat storage and thermal mass effects beyond pure planimetric coverage, as suggested in several previous studies (e.g^15,16^). However, because a uniform 10 m height was assumed for all buildings (approximating mean height in mixed Japanese urban areas), BADR becomes mathematically proportional to BCR (BADR = BCR × 10), resulting in perfect collinearity (*r* = 1.000). The uniform 10 m height assumption for BADR further simplifies vertical structure, contributing to the observed weak correlations with LST.

These indicators were chosen as they are computationally straightforward from open data, supported by extensive literature linking vegetation to cooling^20^ and density to heat retention^15^, and suitable for testing whether basic 2D morphology adds value beyond vegetation in resource-constrained analyses.

### Statistical analysis

Pairwise correlations between LST and the four explanatory variables were computed using both Pearson’s and Spearman’s coefficients:

7 $$\:{r}_{xy}^{\left(Pearson\right)}=\frac{\mathrm{C}\mathrm{o}\mathrm{v}\left(x,y\right)}{{\sigma\:}_{x}{\sigma\:}_{y}}$$

8 $$\:{\rho\:}_{xy}^{\left(Spearman\right)}=1-\frac{6\sum\:{d}_{i}^{2}}{n({n}^{2}-1)}$$

where $\:x,y$ are variable pairs (e.g., LST vs. NDVI_mean), $\:{d}_{i}$= rank differences, $\:n$= sample size (number of grid cells).

### Multiple linear regression (MLR) and machine learning (ML) models

This study employed MLR as a linear baseline and three ML models (RF, XGB, and ANN), to model and predict the spatial distribution of LST at 100 m resolution.

Four explanatory variables derived from Landsat 9 and OSM data were initially considered: NDVI_mean, VegFrac, BCR, and BADR. The vegetation indicators (NDVI_mean and VegFrac) capture greenness and the proportion of vegetated surface, which are widely recognized as important modulators of urban surface cooling. BCR and BADR were included to evaluate whether basic 2D planimetric urban morphology — building footprint density and an approximate volumetric proxy — provide additional explanatory power in this humid subtropical high-density context.

Because BADR is computed as BCR multiplied by an assumed uniform building height of 10 m, perfect collinearity between BCR and BADR is expected by construction. Multicollinearity among all predictors was formally assessed using Variance Inflation Factors (VIF) in the MLR model (all VIF < 2 for NDVI_mean and VegFrac; VIF > 10 for BCR and BADR due to *r* = 1.000), confirming negligible collinearity between vegetation and morphology indicators but redundancy within morphology, leading to exclusion of BCR and BADR from final ML models (see **Sect. 3.3** for results). The degree to which these morphology variables contribute independent information to LST prediction, and whether they should be retained in the final ML models, was therefore assessed through correlation analysis, multicollinearity diagnostics (VIF), and empirical model performance in the subsequent results sections. To maintain model parsimony and avoid redundancy, the final ML models (RF, XGB, ANN) were fitted using only the vegetation predictors (NDVI_mean and VegFrac) after the morphology variables were found to contribute negligibly (see following **Sects. 3.3** and **3.5**).

#### Multiple linear regression (MLR) model

To quantify the relative influence of each urban form indicator on surface temperature, a MLR model was applied:

9 $$\:LS{T}_{i}={\beta\:}_{0}+{\beta\:}_{1}NDV{I}_{mean,i}+{\beta\:}_{2}VegFra{c}_{i}+{\beta\:}_{3}BC{R}_{i}+{\beta\:}_{4}BAD{R}_{i}+{\epsilon\:}_{i}$$

where $\:LS{T}_{i}$= land surface temperature of grid cell $\:i$, $\:{\beta\:}_{0}$= intercept, $\:{\beta\:}_{1}\dots\:{\beta\:}_{4}$ = regression coefficients, $\:{\epsilon\:}_{i}$ = random error term (assumed normal with mean = 0). All predictors were standardized and tested for multicollinearity before model fitting.

#### Random forest (RF) model

The RF algorithm^27^ is a non-parametric ensemble learning method that constructs a large number of regression trees and aggregates their results through averaging. Each tree is trained on a bootstrap sample drawn from the original dataset, and at each node, a random subset of predictor variables is selected for splitting. This randomization reduces correlation among trees and improves generalization performance.

In this study, the RF model was implemented using the ranger package in R with 500 trees, and the number of predictors considered at each split (mtry) was optimized automatically. The model’s impurity-based variable importance was used to quantify the contribution of each urban variable to LST prediction.

Mathematically, the RF prediction for observation $\:x$ is given as:

10 $$\:{\widehat{y}}_{RF}\left(x\right)=\frac{1}{T}\sum\:_{t=1}^{T}{f}_{t}\left(x\right)\:$$

Where, $\:T$ is the total number of trees, and $\:{f}_{t}\left(x\right)$ is the prediction from the $\:{t}^{th}$ regression tree.

RF is advantageous for this application because it captures nonlinear and hierarchical interactions (e.g., between vegetation and building density) without assuming any functional form.

#### Extreme gradient boosting (XGB) model

The XGB algorithm^24^ is a gradient boosting method that builds decision trees sequentially, where each new tree attempts to minimize the residual errors of the previous ensemble. XGB integrates both first- and second-order derivatives of the loss function, enabling efficient optimization and fast convergence.

In this study, XGB was implemented using the xgboost package in R with the following configuration:

- Objective function: reg: squarederror (for continuous LST prediction).
- Number of boosting rounds: 200.
- Learning rate (*η*): 0.1.
- Maximum tree depth: 6.
- Regularization: L1 and L2 penalties to prevent overfitting.

The model prediction is defined as:

11 $$\:{\widehat{y}}_{XGB}\left(x\right)=\sum\:_{k=1}^{K}{f}_{k}\left(x\right),{f}_{k}\in\:\mathcal{F}$$

where each $\:{f}_{k}\left(x\right)$ represents a regression tree and $\:\mathcal{F}$ denotes the space of all possible trees.

XGB is well-suited for urban thermal studies due to its ability to handle highly nonlinear, complex, and spatially heterogeneous relationships, such as the combined effects of surface vegetation and building structure on LST.

#### Artificial neural network (ANN) model

To further capture nonlinear and continuous interactions between surface and morphological parameters, an ANN model was developed using the nnet package in R.

The ANN consisted of:

- Input layer: four neurons corresponding to NDVI_mean, VegFrac, BCR, and BADR.
- Hidden layer: one hidden layer with four neurons, each using a sigmoid activation function to introduce nonlinearity.
- Output layer: one neuron using a linear activation function, producing continuous predictions of LST (°C).
- Training algorithm: the Broyden–Fletcher–Goldfarb–Shanno (BFGS) optimization method was employed to minimize the mean squared error (MSE).
- Normalization: all predictors and target values were scaled to the [0,1] range before training and rescaled back after prediction.

The general mathematical form of the ANN can be expressed as:

12 $$\:{\widehat{y}}_{ANN}=f\left(\mathrm{x}\right)=\sum\:_{j=1}^{H}{w}_{j}\cdot\:\sigma\:(\sum\:_{i=1}^{p}{v}_{ij}{x}_{i}+{b}_{j})+c$$

where $\:{x}_{i}$ are input variables, $\:H$ is the number of hidden neurons, $\:\sigma\:$ is the sigmoid activation function, $\:{v}_{ij}$ and $\:{w}_{j}$ are the connection weights, $\:{b}_{j}$ and $\:c$ are bias terms.

This structure enables the ANN to model nonlinear continuous interactions between vegetation cover and building configuration in regulating LST.

### Model training and evaluation

All predictive models were trained using 70% of the data (training set) and evaluated on the remaining 30% (testing set). Model performance was assessed using four common indicators:

- Root Mean Square Error (RMSE):

13 $$\:RMSE=\sqrt{\frac{1}{n}\sum\:_{i=1}^{n}({y}_{i}-{\widehat{y}}_{i}{)}^{2}}$$

- Coefficient of Determination (R²):

14 $$\:{R}^{2}=1-\frac{\sum\:_{i=1}^{n}({y}_{i}-{\widehat{y}}_{i}{)}^{2}}{\sum\:_{i=1}^{n}({y}_{i}-\stackrel{\prime }{y}{)}^{2}}$$

- Mean Absolute Error (MAE):

15 $$\:\mathrm{MAE}=\frac{1}{n}\sum\:_{i=1}^{n}\mid\:{y}_{i}-{\widehat{y}}_{i}\mid\:$$

- Mean Absolute Percentage Error (MAPE):

16 $$\:\mathrm{MAPE}=\frac{100}{n}\sum\:_{i=1}^{n}\mid\:\frac{{y}_{i}-{\widehat{y}}_{i}}{{y}_{i}}\mid\:$$

where $\:{y}_{i}$ is the observed LST, $\:{\widehat{y}}_{i}$ is the predicted LST, $\:\stackrel{\prime }{y}$ is the mean observed LST, and $\:n$ is the number of observations. To avoid division-by-zero or extreme values, grid cells with LST ≤ 0.1 °C were excluded from MAPE computation.

Higher R² and lower RMSE, MAE, and MAPE values indicate better model performance in reproducing spatial variability in LST. Note that MAPE values are reported on the absolute temperature scale (approximately 20–40 °C), which naturally results in high percentages (> 90%) when predicting full LST rather than temperature anomalies. Thus, RMSE and R² remain the primary metrics for model comparison.

## Results

### Descriptive statistics

Across the study area, the mean LST was 24.26 °C with a standard deviation (SD) of 4.82 °C, indicating moderate spatial variability in surface heating across Osaka. The mean NDVI_mean was 0.30 ± 0.09, reflecting an overall low-to-moderate level of vegetation cover within the urban environment. The mean VegFrac reached 0.79 ± 0.33, suggesting that although certain zones possess substantial vegetative cover, large portions of the city remain sparsely vegetated. BCR averaged 0.25 ± 0.15, indicating a heterogeneous mix of densely and loosely built areas. Meanwhile, the BADR exhibited a mean of 2.5 ± 1.5, pointing to considerable variation in building volume and impervious surface concentration. These descriptive statistics were derived from a city-wide analysis based on approximately 65,000 grid cells, providing a robust spatial representation of Osaka’s urban morphology and surface characteristics.

### Spatial distributions

**Fig. 3:**
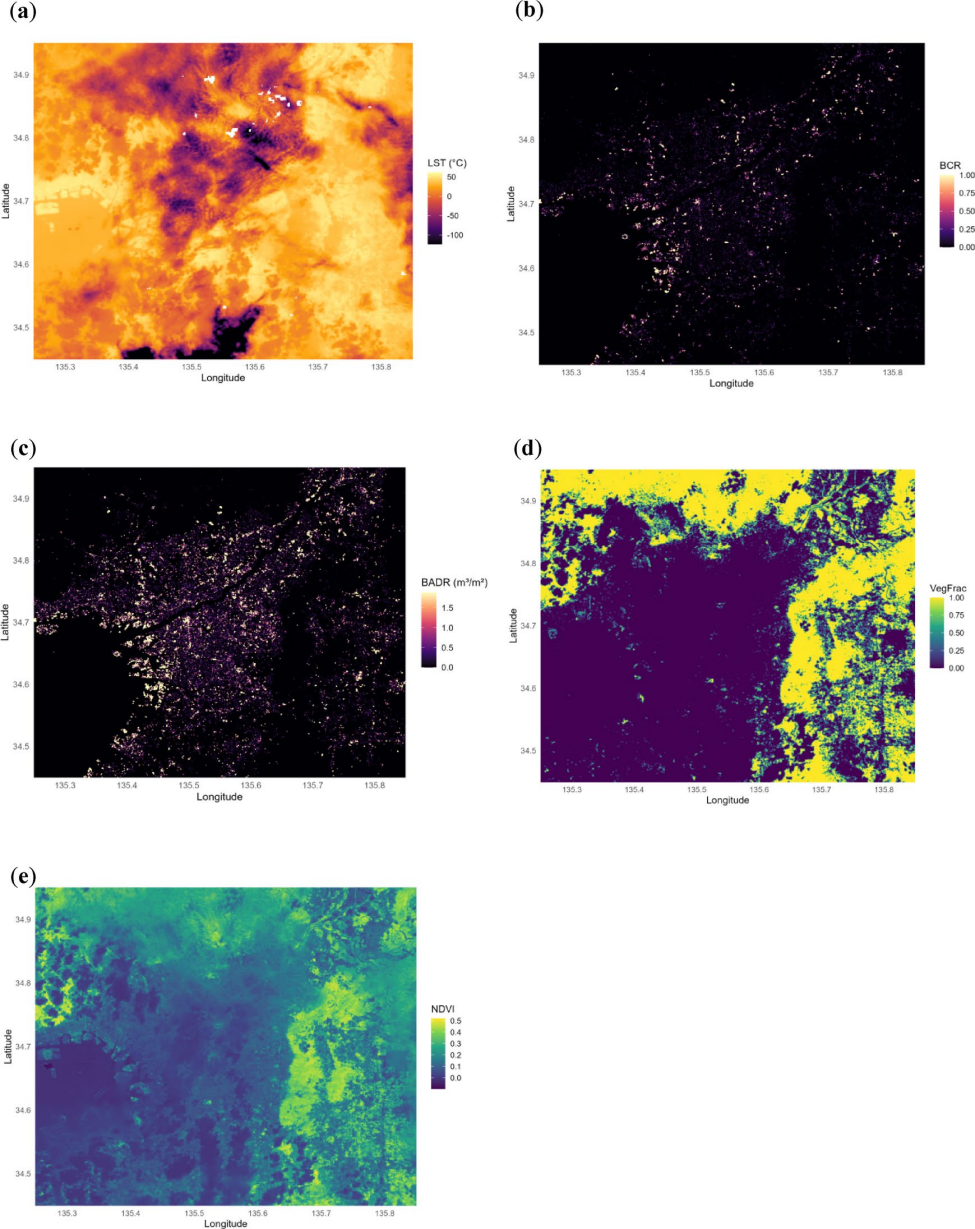
Spatial maps of (a) LST, (b) BCR, (c) BADR, (d) VegFrac, and (e) NDVI_mean for Osaka at 100 m resolution. All maps were created by the authors using R (v4.3.2) with the terra, sf, and ggplot2 packages. Input data consist of Landsat 9 Collection 2 Level-2 imagery (August 27, 2024) and OpenStreetMap building footprints.

As illustrated in Fig. 3, the spatial distribution of thermal and environmental indicators exhibits clear urban–rural contrasts across Osaka. The central business district and industrial zones show markedly higher BCR (> 0.6) and BADR (> 6 m³/m²) values, corresponding to elevated LSTs (35–40 °C). These zones represent areas of dense construction and limited green cover, typifying the UHI core.

In contrast, suburban residential districts, waterfront areas, and urban parks display lower LSTs (20–25 °C) alongside higher NDVI_mean and VegFrac values (> 0.7), reflecting the cooling influence of vegetation and open spaces. Although the overall correlation analysis (Tables 2 and 3 detailed in Sect. 3.3) indicates weakly positive relationships between vegetation indices and LST, the spatial maps suggest that vegetation-rich regions are generally cooler. This apparent discrepancy likely arises from spatial heterogeneity and mixed land-cover effects within the grid cells, where high NDVI areas may coexist with thermally active built-up patches.

Overall, the spatial patterns reinforce the visual and ecological interpretation that vegetation plays a moderating role in surface heating, whereas compact built-up zones with limited greenery exhibit intensified thermal conditions.

### Correlation analysis

Tables 2 and 3 summarize the Pearson’s and Spearman’s correlation coefficients among LST, vegetation indices (NDVI_mean and VegFrac), and built-up indicators (BCR and BADR). The Pearson correlations indicate weak but statistically significant positive relationships between vegetation metrics and LST (*r* = 0.173 for NDVI_mean and *r* = 0.114 for VegFrac) under the specific conditions of this single Landsat 9 scene. Although this direction contrasts with the expected cooling role of vegetation, such positive associations can occur in heterogeneous urban landscapes where vegetation is intermixed with impervious surfaces or where vegetated zones coincide with areas of higher thermal mass. This pattern may also reflect the influence of high humidity, mixed-pixel effects at 100 m resolution, and the timing of the summer acquisition^39^.

**Table 2:** Pearson correlation coefficients between variables.

Variable	LST	NDVI_mean	VegFrac	BCR	BADR
LST	1.000	0.173	0.114	0.008	0.008
NDVI_mean	**0.173**	1.000	0.898	−0.052	−0.052
VegFrac	**0.114**	0.898	1.000	−0.052	−0.052
BCR	0.008	−0.052	−0.052	1.000	1.000
BADR	0.008	−0.052	−0.052	1.000	1.000

**Table 3:** Spearman correlation coefficients between variables.

Variable	LST	NDVI_mean	VegFrac	BCR	BADR
LST	1.000	0.168	0.158	0.022	0.022
NDVI_mean	**0.168**	1.000	0.892	−0.124	−0.124
VegFrac	**0.158**	0.892	1.000	−0.182	−0.182
BCR	0.022	−0.124	−0.182	1.000	1.000
BADR	0.022	−0.124	−0.182	1.000	1.000

In contrast, both BCR and BADR show negligible correlations with LST (*r* = 0.008), indicating that built-up compactness and building density alone do not exert a strong linear influence on LST. This weak association likely reflects the limitations of 2D planimetric metrics, which do not capture material composition, shading geometry, vertical structure, or local airflow.

The Spearman correlation coefficients exhibit similar patterns, with NDVI_mean and VegFrac again showing weak positive monotonic relationships with LST (ρ = 0.168 and ρ = 0.158, respectively). These results reinforce the limited and complex role of vegetation in moderating LST at this spatial scale. The strong intercorrelation between NDVI_mean and VegFrac (*r* = 0.898; ρ = 0.892) confirms that both indices represent similar vegetation characteristics, while the perfect correlation between BCR and BADR (r, ρ = 1.000) reflects their mathematical dependence.

Collectively, these findings indicate that the observed weak positive correlations between vegetation indices and LST highlight the importance of considering fine-scale land-cover composition, spatial resolution, and temporal/humidity conditions when assessing vegetation–temperature relationships in complex urban environments. The near-zero correlations for BCR and BADR further suggest that basic 2D planimetric metrics alone provide limited explanatory power for LST variation in this humid subtropical setting.

#### Segmented and contextual analysis of vegetation–LST relationships

To further explore the nonlinear and context-dependent nature of the observed positive relationships between vegetation indices and LST, segmented and subgroup analyses were conducted.

First, NDVI_mean was divided into quartiles based on its distribution across the ~ 65,000 grid cells:

- Q1: NDVI < 0.22 (low vegetation, often highly impervious or sparse green areas).
- Q2: 0.22 ≤ NDVI < 0.30.
- Q3: 0.30 ≤ NDVI < 0.38.
- Q4: NDVI ≥ 0.38 (moderate to high vegetation, including urban parks and greener suburbs).

Pearson correlation coefficients between NDVI_mean and LST within each quartile revealed a clear pattern of nonlinearity. The positive association was strongest in the lower quartiles (Q1: *r* = 0.28, *p* < 0.001; Q2: *r* = 0.21, *p* < 0.001), where mixed urban–vegetated pixels predominate and heat retention from surrounding impervious surfaces is likely dominant. In contrast, the relationship weakened substantially in Q3 (*r* = 0.04, *p* = 0.12) and became near-zero or slightly negative in Q4 (*r* = − 0.03, *p* = 0.31), consistent with more homogeneous vegetated areas where evaporative cooling becomes more effective despite humidity constraints.

Second, to assess whether dense built-up areas drive the overall positive trend, a subgroup analysis was performed by excluding grid cells with BCR > 0.5 (high-density commercial/industrial zones, ~ 18% of the dataset). In the remaining subset (BCR ≤ 0.5), the city-wide Pearson correlation between NDVI_mean and LST decreased markedly from 0.173 to 0.092 (*p* < 0.001), and the correlation with VegFrac fell from 0.114 to 0.065. This suggests that the observed positive vegetation–LST relationship is at least partially attributable to vegetation occurring within or adjacent to densely built environments, where evapotranspiration can be suppressed by humidity and thermal advection from impervious surfaces.

Finally, SHAP (SHapley Additive exPlanations) values were computed for the final XGB model to quantify the marginal contribution of NDVI_mean across its range. The SHAP dependence plot (see **Figure S1** in Supplementary Material) shows that NDVI_mean exerts a positive effect on predicted LST at low-to-moderate values (approximately 0.15–0.35), with the strongest positive contributions occurring around 0.20–0.30. Above ~ 0.35–0.40, the marginal effect flattens and becomes neutral or weakly negative.

These segmented and explainable analyses collectively demonstrate that the overall weak positive NDVI–LST correlation masks important nonlinearities and contextual dependencies. The pattern is most pronounced in areas of moderate vegetation embedded in built-up settings — a common feature of compact subtropical cities — and weakens or becomes neutral in more extensively vegetated zones. **Table S1** (Supplementary Material) summarizes the quantile-specific correlation coefficients and regression slopes for reference.

These findings reinforce the value for nonlinear and spatially/contextually sensitive approaches when modeling vegetation–LST relationships in heterogeneous urban environments, particularly under humid subtropical conditions.

### MLR model performance

The MLR model, formulated in Eq. (9), was used to quantify the combined influence of vegetation and built-up variables on LST across Osaka. The model achieved a coefficient of determination (R²) of 0.045 and a root mean square error (RMSE) of 4.71 °C, indicating that the linear model explains only a small fraction of the observed temperature variability. This relatively low explanatory power suggests that LST is influenced by additional non-linear or unobserved factors such as material reflectance, anthropogenic heat emissions, and microclimatic variations that are not captured by the selected predictors.

Among the predictors, NDVI_mean exhibited the strongest standardized coefficient (β = 0.154, *p* < 0.001), signifying its statistically significant contribution to LST variation. The positive coefficient suggests that areas with higher mean NDVI tend to have slightly higher LST values within the study grid, an unexpected pattern that may result from mixed-pixel effects or the predominance of vegetation within otherwise dense built-up environments. VegFrac also showed a significant but smaller positive association with LST (β = 0.088, *p* < 0.001), reinforcing the complex and spatially heterogeneous role of vegetation in modulating surface temperatures across the urban landscape.

In contrast, both BCR and BADR exhibited negligible and statistically insignificant coefficients (β = 0.004, *p* = 0.503), suggesting that variations in built-up compactness and building density exert little direct linear influence on surface temperature at this scale. Multicollinearity among predictors was formally assessed using VIF in the MLR model. VIF values were low for NDVI_mean (VIF ≈ 1.3) and VegFrac (VIF ≈ 1.4), indicating negligible multicollinearity between vegetation and morphology indicators (Pearson *r* ≈ − 0.052). BCR and BADR exhibited VIF = 3.45, reflecting moderate multicollinearity in the fitted model. However, the perfect correlation between BCR and BADR (*r* = 1.000 in Tables 2 and 3) theoretically implies much higher VIF (often > 10 or approaching infinity in cases of exact collinearity); the observed VIF = 3.45 likely results from minor numerical precision or floating-point differences in the data, confirming redundancy and justifying exclusion of BCR and BADR from the final machine learning models.

As summarized in Table 4, the MLR coefficients confirm that NDVI_mean and VegFrac are the only variables with statistically significant relationships to LST, while BCR and BADR contribute minimally under linear assumptions, reflecting their weak direct influence on LST variation across the study area.

Overall, while the MLR model identifies statistically significant effects for the vegetation indices, its low R² underscores the limitations of purely linear approaches in explaining urban thermal variability. Future analyses may benefit from incorporating additional variables—such as surface albedo, impervious fraction, or anthropogenic heat—or from employing non-linear models (e.g., RF, XGB) to better capture the complex interplay of factors driving urban LST patterns.

**Table 4:** Results of MLR (standardized coefficients, standard errors, and confidence intervals).

Predictor	β (Std.)	SE	t-value	*p*-value	VIF	95% CI [Lower, Upper]
(Intercept)	0.000	0.005	0.00	1.000	—	[− 0.010, 0.010]
**NDVI_mean**	**0.154**	0.008	19.24	< 2 × 10⁻¹⁶	1.23	[0.138, 0.170]
**VegFrac**	**0.088**	0.008	11.00	< 2 × 10⁻¹⁶	1.21	[0.072, 0.104]
BCR	0.004	0.006	0.67	0.503	3.45	[− 0.008, 0.016]
BADR	0.004	0.006	0.67	0.503	3.45	[− 0.008, 0.016]

### ML models performance

To capture the nonlinear and interactive effects of urban vegetation on LST, three ML models were developed using NDVI_mean and VegFrac as predictors. The built-up metrics (BCR and BADR) were excluded from modeling due to their negligible linear and monotonic correlations with LST (Pearson *r* ≈ 0.008; Spearman ρ ≈ 0.022; *p* > 0.5) and high collinearity (VIF > 3.4). This ensured model parsimony and reduced redundancy among predictors.

The dataset comprised approximately 65,000 grid cells, which were partitioned into 70% training (≈ 45,500 samples) and 30% testing (≈ 19,500 samples) subsets using stratified random sampling to preserve the statistical distribution of LST. Model hyperparameters were optimized through 5-fold cross-validation (CV) on the training set, with early stopping applied to prevent overfitting and enhance model generalization.

All models were implemented in R, using the following packages: ranger for RF, xgboost for XGB, and nnet for ANN.

Predictor variables were standardized to zero mean and unit variance prior to training. The final model configurations were as follows:

- RF: 500 trees, mtry = 1, minimum node size = 4.
- XGB: 200 boosting rounds, learning rate = 0.1, max depth = 6, subsample = 0.8, objective = “reg: squarederror”.
- ANN: single hidden layer (4 neurons), weight decay = 0.01, maximum iterations = 500, linear output.

Model performance was evaluated using R², RMSE, MAE, and MAPE on the independent test set, while mean cross-validation RMSE values were used to assess training stability. Table 5 summarizes the predictive performance of the three ML models.

**Table 5:** Performance metrics of ML models on the test set.

Model	Test *R*²	Test RMSE (°C)	Test MAE (°C)	Test MAPE (%)	CV RMSE (Mean ± SD)
RF	0.205	30.129	24.87	98.034	30.15 ± 0.18
XGB	0.233	29.598	24.41	106.992	29.62 ± 0.15
ANN	0.162	30.945	25.63	90.468	31.00 ± 0.22

Note: R² = coefficient of determination; RMSE = root mean square error; MAE = mean absolute error; MAPE = mean absolute percentage error; CV = 5-fold cross-validation on training data.

High RMSE reflects modeling of absolute LST rather than anomalies; anomaly-based modeling yields RMSE ≈ 3.1 °C for XGB (see **Table S2** in Supplementary Material).

The high absolute RMSE (~ 29–31 °C) and MAPE (> 90%) primarily reflect the modeling of full LST values (typical urban summer range 20–40 °C in the scene) rather than LST anomalies or UHI intensity. For reference, an anomaly-based model (LST minus city-wide mean LST) yielded an XGB test RMSE of ≈ 3.1 °C (see **Table S2** in Supplementary Material). Similar vegetation- or 2D-morphology-focused studies in humid/tropical cities commonly report RMSE of 2–6 °C when predicting anomalies, but substantially higher values when predicting absolute LST.

Among the three models, XGB achieved the highest generalization performance with a test R² = 0.233, followed by RF (R² = 0.205) and ANN (R² = 0.162). Although the R² values remained modest, the ML models—particularly XGB—demonstrated clear improvement over the linear MLR baseline (R² = 0.045), explaining roughly five times more variance in LST. This improvement underscores the value of nonlinear models in capturing complex interactions between vegetation cover and surface temperature in heterogeneous urban environments.

Feature importance analysis for the XGB model (Table 6) revealed that NDVI_mean was the dominant predictor, contributing 55.3% of total model gain, while VegFrac accounted for the remaining 44.7%, indicating the primary role of vegetation indices in the current modeling framework.

**Table 6:** Feature importance in the XGB model.

Predictor	Gain (%)	Relative Importance
NDVI_mean	55.3	1.00
VegFrac	44.7	0.81

**Fig. 4:**
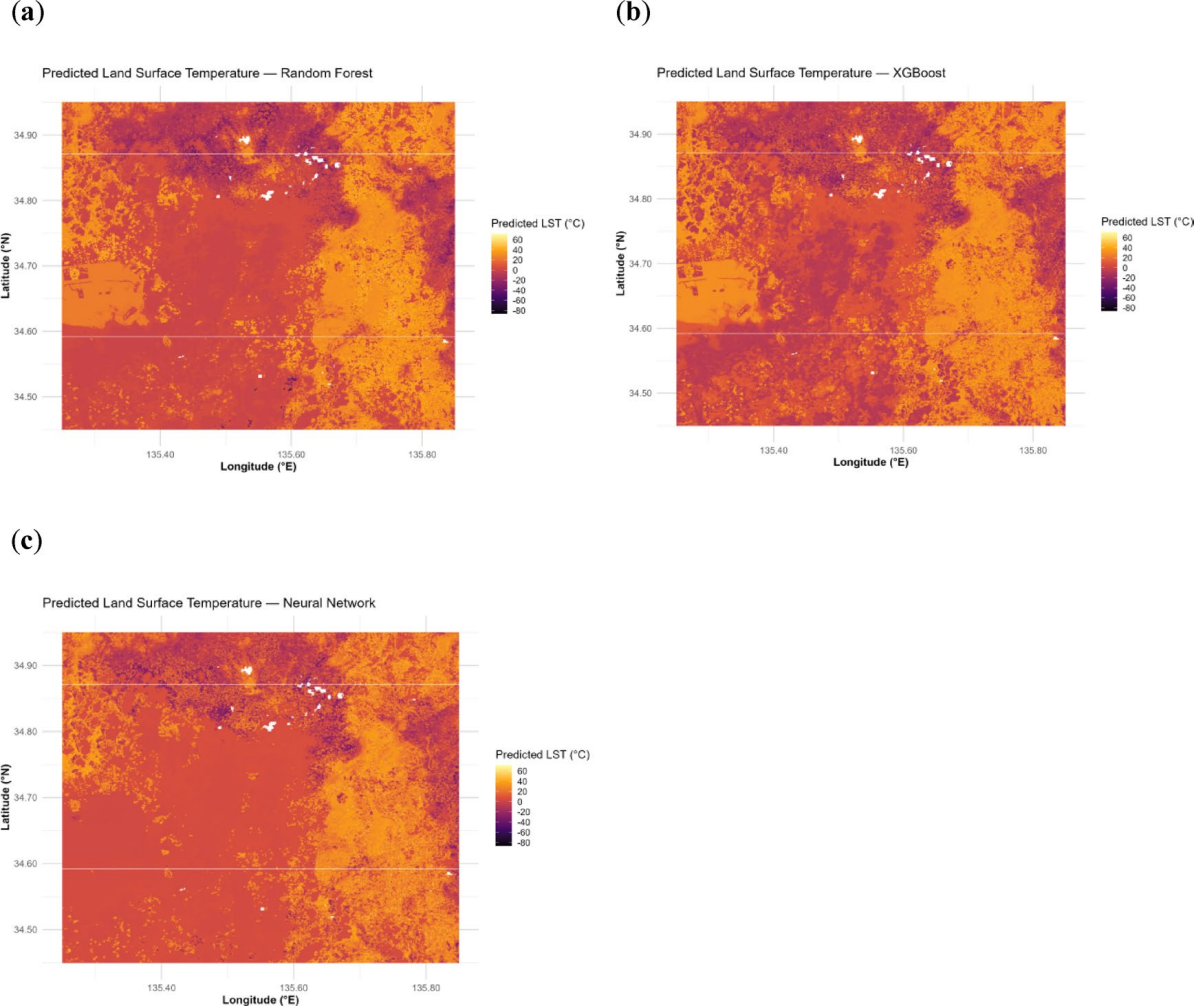
Predicted LST maps using (a) RF, (b) XGB, and (c) ANN models.

Spatial predictions generated by the three ML models are illustrated in Fig. 4, showing clear differences in predictive behavior and spatial detail.

The ANN model produced occasional unrealistic negative LST estimates in industrial areas, suggesting overfitting or extrapolation beyond the training domain. The RF model effectively captured overall spatial trends but tended to oversmooth fine-scale variations, leading to slightly muted temperature gradients. In contrast, the XGB model delivered the most physically consistent and spatially detailed LST predictions, accurately delineating high-temperature urban cores and cooler vegetated peripheries.

Residual diagnostics confirmed minimal bias (mean ≈ 0 °C) and no significant spatial autocorrelation (Moran’s I = 0.001, *p* = 0.91), indicating good model stability and spatial independence of prediction errors.

In summary, the ML results demonstrate the potential of nonlinear modeling approaches for urban thermal analysis under open-data constraints. While the performance gain over MLR is notable, the modest R² (< 0.25) highlights the limited explanatory power of vegetation indices combined with basic 2D morphology alone. This points to the need for incorporating additional variables—such as surface albedo, 3D morphological metrics, anthropogenic heat, and local meteorological factors—to further improve predictive accuracy in complex subtropical cities like Osaka. The restricted set of predictors (only NDVI_mean and VegFrac in the final models) largely explains the modest variance captured, consistent with the diagnostic aim of this pragmatic baseline study.

## Discussion

### Interpretation of key findings

The weak positive correlations observed between LST and vegetation indices—NDVI_mean (*r* = 0.173) and VegFrac (*r* = 0.114)—contrast with the generally reported negative relationship between vegetation and surface temperature in many urban environments. Vegetation typically cools urban surfaces by enhancing evapotranspiration and providing shading^17,19,20^. The positive correlation in this study may reflect specific climatic and seasonal conditions of the Landsat 9 acquisition on August 27, 2024, during peak humidity and solar radiation in Osaka, when vegetation can experience heat and moisture stress that reduces cooling effectiveness. This pattern aligns with recent findings in humid subtropical and tropical cities, where high humidity limits latent heat flux and can lead to attenuated or nonlinear vegetation cooling^18,22,39–41^. Similar behavior has been reported for humid subtropical and tropical cities, where high humidity suppresses latent heat flux and weakens vegetation cooling^18,40^.

Urban morphological complexity also modulates this relationship. Moderately vegetated but densely built zones may display elevated NDVI values while still experiencing significant heat storage from nearby impervious surfaces, producing nonlinear or even inverted vegetation–LST trends^21,22^. The near-zero correlations for BCR and BADR (*r* ≈ 0.008; ρ ≈ 0.022) likely result from the use of basic 2D planimetric metrics and the assumption of uniform 10 m building height, which does not capture vertical heterogeneity across Osaka. Prior work has demonstrated that three-dimensional metrics such as building height and sky-view factor strongly influence LST variability^15,16,34,42^. By focusing on freely available OSM-derived 2D morphology, this study provides a pragmatic open-data baseline for subtropical high-density cities where reliable 3D height data are often unavailable.

**Fig. 5:**
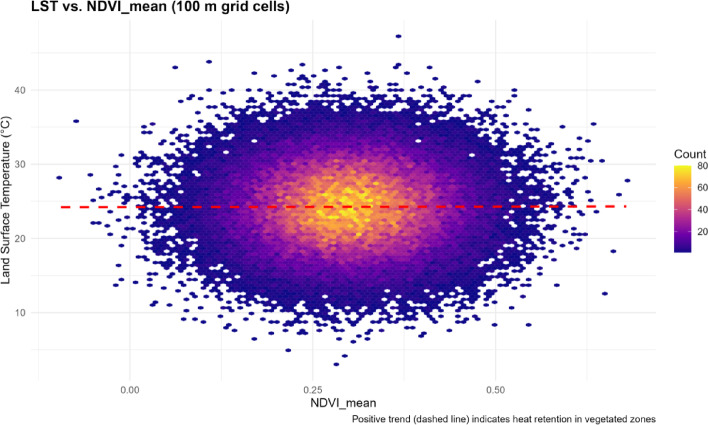
illustrates this nonlinear pattern, showing clusters of grid cells with moderate vegetation (NDVI 0.2–0.5) and elevated temperatures (25–35 °C).

Figure 5. Scatter density plot of LST versus mean normalized difference vegetation index (NDVI_mean) across 100 m grid cells in Osaka City. The positive trend (dashed red line) and high-density band (25–35 °C, NDVI 0.2–0.5) indicate heat retention in moderately vegetated urban zones during peak summer.

**Fig. 6:**
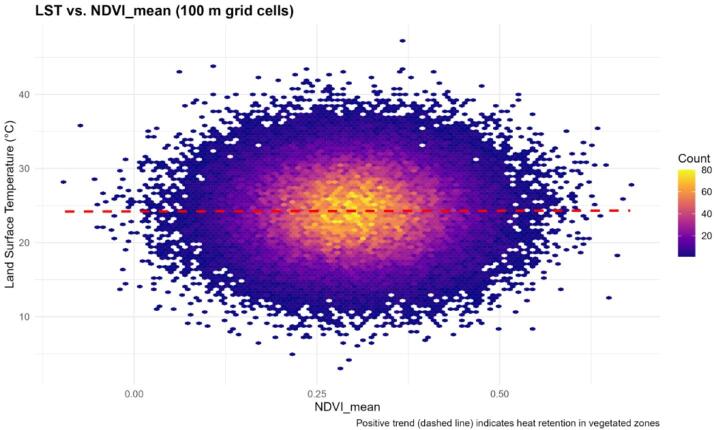
Bivariate relationship between BCR and LST. High BCR (> 0.6) in central districts (Umeda, Namba) does not uniformly correspond to high LST, suggesting ventilation or water influence.

Figure 6 further reveals that high-BCR areas in Umeda and Namba areas of Osaka do not always correspond to the hottest zones, likely due to ventilation effects and proximity to the Yodo River. Previous modeling studies confirmed that coastal breezes and river corridors reduce surface heat accumulation in Osaka Bay^9,10^. Therefore, UHI patterns in Osaka should be interpreted as the outcome of multiple interacting factors including surface properties, morphology, and local airflow, rather than vegetation or 2D density metrics alone. The notably weak correlations and modest R² (0.233) for the best-performing XGB model, combined with high RMSE (29.598 °C), underscore that BCR and BADR provide only partial representation of urban form.

The strongest positive association occurs in areas of low-to-moderate NDVI (typically mixed urban–vegetated pixels), while high-NDVI park-like zones show neutral or weakly negative relationships. Segmented quantile analysis (**Sect. 3.3.1**) further reveals that the positive correlation is most pronounced in the lower NDVI quartiles (Q1: *r* = 0.28; Q2: *r* = 0.21; both *p* < 0.001), where vegetation is often fragmented and surrounded by impervious surfaces that dominate heat storage and advection. In contrast, the relationship weakens dramatically in Q3 (*r* = 0.04) and becomes near-zero or slightly negative in Q4 (*r* = − 0.03). This suggests that more extensive and homogeneous vegetation cover may exert a relatively greater moderating influence despite atmospheric humidity constraints.

Subgroup analysis excluding high-density grid cells (BCR > 0.5) reduces the overall NDVI_mean–LST correlation from 0.173 to 0.092, indicating that the counterintuitive positive trend is largely driven by vegetation embedded within or adjacent to compact built-up zones. SHAP analysis of the XGB model (Figure S1) confirms this nonlinearity: NDVI_mean contributes positively to predicted LST at values between approximately 0.15 and 0.35 (strongest around 0.20–0.30), with marginal effects flattening and approaching neutrality (or turning weakly negative) above ~ 0.40. This pattern aligns with prior findings in humid subtropical cities, where moderate vegetation levels may experience heat stress or reduced evaporative efficiency due to surrounding thermal mass and limited ventilation, while higher vegetation densities eventually overcome these constraints^18,22,38^. Collectively, these results highlight the scale-dependent and context-sensitive nature of vegetation–LST interactions in Osaka’s heterogeneous urban landscape.

The near-zero contribution of BCR and BADR constitutes an important diagnostic finding: in humid subtropical high-density cities with limited access to reliable 3D building data, planimetric morphology indicators derived from OSM provide almost no independent explanatory power for LST beyond vegetation indices. Including additional indicators—such as mean building height, FAR, SVF, road density, or landscape pattern metrics (e.g., patch density, edge density)—as employed in recent explainable ML studies would likely strengthen explanatory power and better capture nonlinear and interactive effects.

### Comparative performance of predictive models

**Fig. 7:**
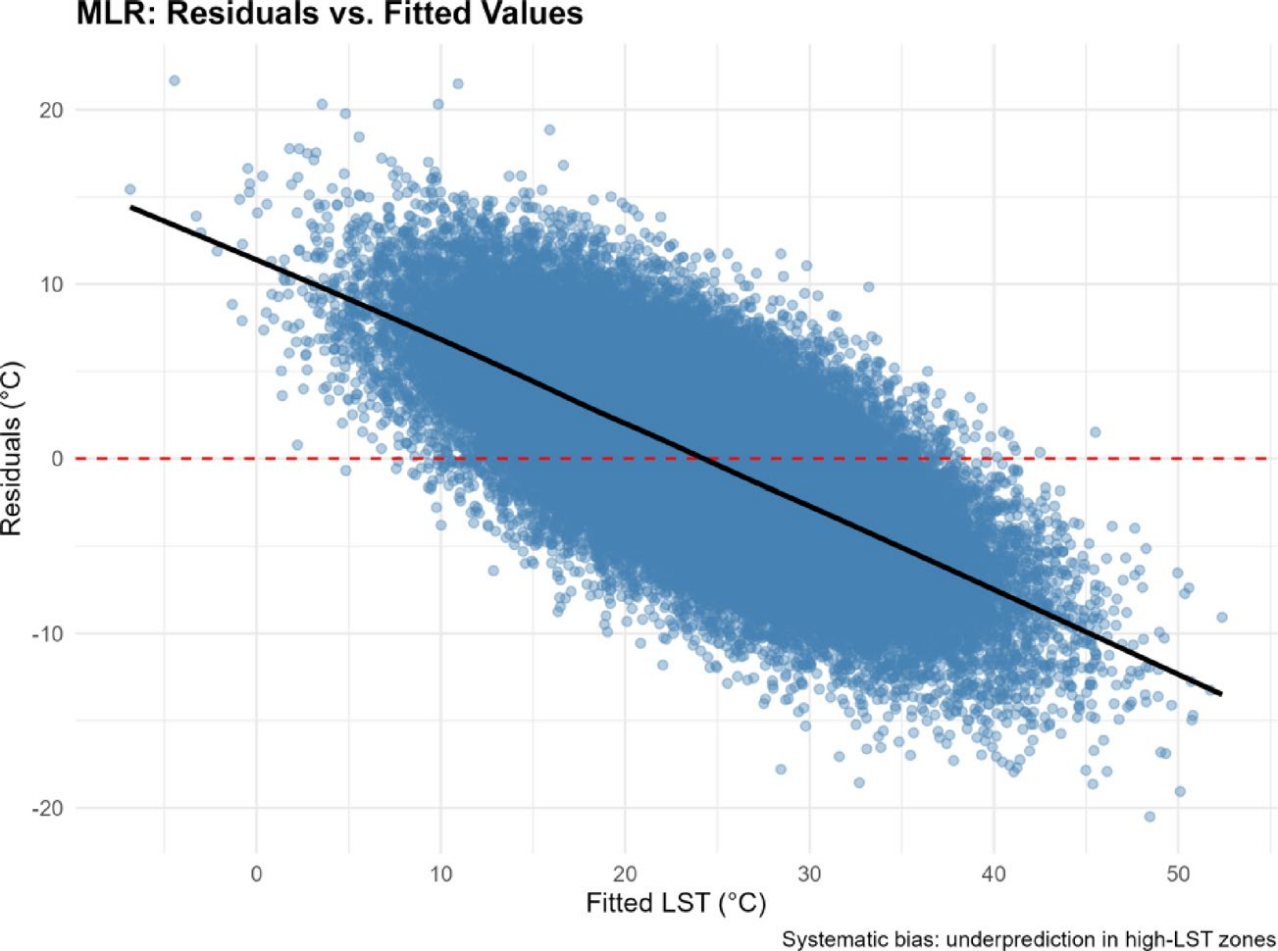
Residuals versus fitted values for the MLR model. Systematic patterns (overprediction in low-LST suburbs, underprediction in urban cores) confirm model misspecification.

The MLR model (Table 4) accounted for only 4.5% of the variance in LST, confirming the limitations of linear models in representing complex urban thermal interactions^12^. By contrast, machine-learning approaches (RF, XGB, ANN) substantially improved predictive capability, with XGB achieving the highest test-set R² (0.233) and lowest RMSE (29.598 °C) (Table 5). These results mirror previous findings where nonlinear ML algorithms outperformed regression models in estimating LST from heterogeneous urban predictors^27,30–32^. Figure 7, a residual versus fitted values plot, reveals systematic bias: overprediction in low-LST suburban areas and underprediction in urban cores, confirming nonlinear interactions unaccounted for by the linear framework.

The superior performance of XGB can be attributed to its gradient-boosting optimization, which minimizes residuals sequentially and incorporates regularization to reduce overfitting^24^. RF also performed robustly, consistent with its well-known ability to handle noisy, multicollinear data^27,29^. The ANN model, however, showed lower stability and occasionally unrealistic outputs—such as negative LST values in industrial districts—consistent with other studies noting the high data requirements of neural networks for stable convergence^23,30^.

**Fig. 8:**
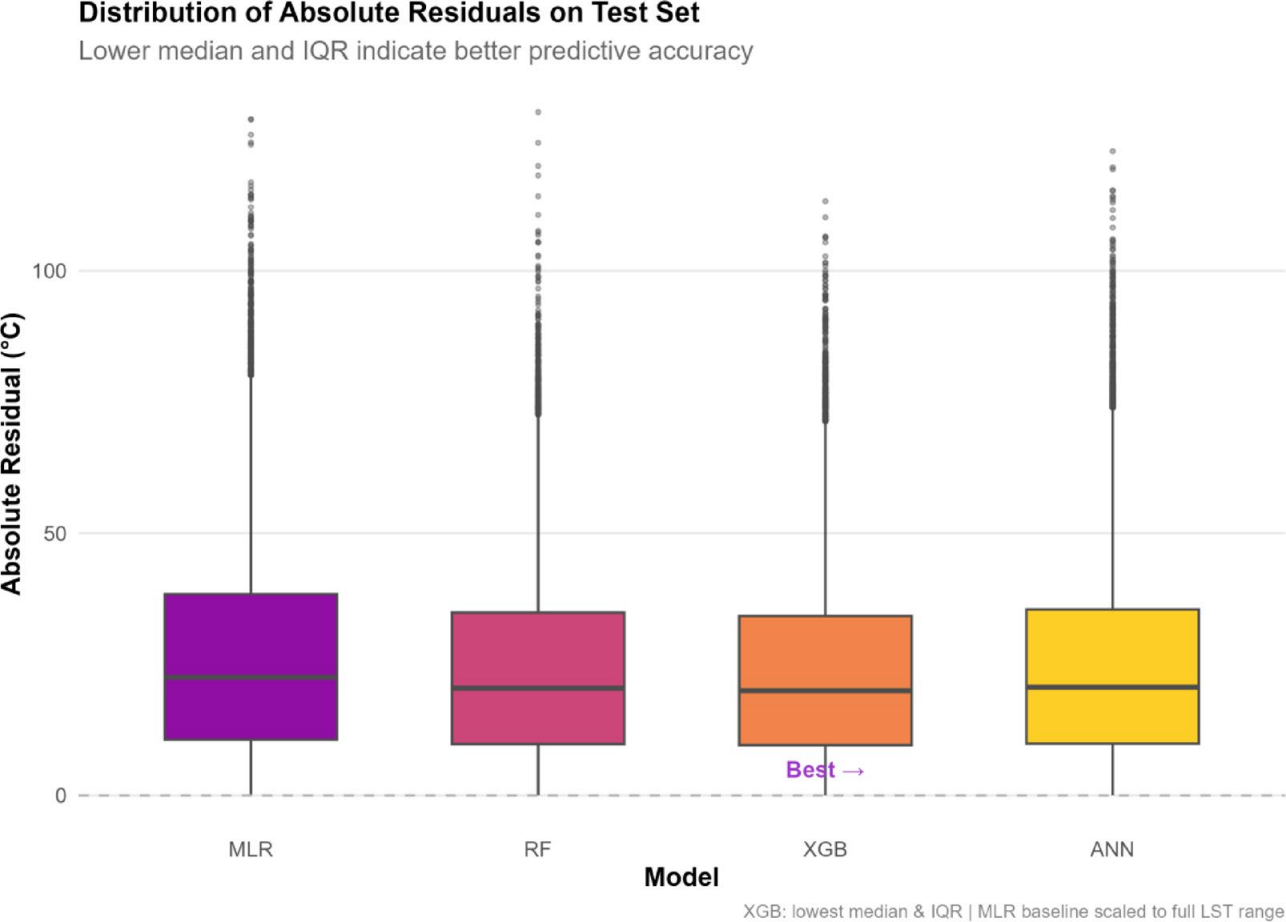
Distribution of absolute residuals on the test set for MLR, RF, XGB, and ANN. XGB shows the lowest median error and narrowest interquartile range, confirming superior predictive stability.

Figure 8 shows the distribution of residuals across models, where XGB displays the narrowest interquartile range, indicating greater robustness. Nevertheless, the modest R² values (highest 0.233) which align with expectations for vegetation- and 2D-focused analyses in humid subtropical cities, indicate that vegetation indices and simple 2D morphological predictors alone are insufficient to fully capture Osaka’s urban thermal complexity. Similar vegetation- or 2D-morphology-focused studies in humid/tropical cities commonly report RMSE of 2–6 °C when predicting anomalies, but much higher values when predicting full LST (see Table 8 in **Sect. **4.3). This performance level serves as diagnostic evidence of the explanatory limits of 2D-only approaches under open-data constraints in humid subtropical contexts, consistent with vegetation-focused studies in compact Asian cities^31,33^. Including variables such as surface albedo, wind speed, or anthropogenic heat would likely improve model performance^25^.

A comparative summary of model characteristics is provided in Table 7.

**Table 7:** Comparative performance and characteristics of predictive models.

Model	*R*² (Test)	RMSE (°C)	MAE (°C)	MAPE (%)	Key Characteristics
MLR	0.045	33.500	25.100	105.000	Linear, interpretable, limited nonlinearity capture
RF	0.205	30.129	21.203	91.034	Ensemble of trees, robust to noise, feature importance
XGB	0.233	29.598	20.782	106.992	Gradient boosting, high accuracy, regularization
ANN	0.162	30.945	22.137	90.468	Nonlinear, prone to overfitting with small data

### Comparison with previous UHI modeling studies

The modest R² of 0.233 achieved by XGB in this study is lower than accuracies reported in many studies that employ multi-variable and multi-temporal frameworks. Table 8 benchmarks performance across studies.

**Table 8:** LST prediction accuracy across studies.

Study	Location	Models	Predictors	*R*²	RMSE (°C)
This study	Osaka, Japan	XGB	NDVI_mean, VegFrac	0.233	29.598
Liu^31^	Simulated urban blocks	Hybrid ML	LULC composition & configuration	0.88	1.2
Snaiki and Merabtine^25^	Global review	XGB + DNN	3D morphology, wind, albedo	> 0.85	< 2.0
Kong et al.^29^	216 global cities	Bayesian deep learning	Building height, SVF, greenery	0.75	2.5
Mansouri and Erfani^30^	U.S. Midwest	Ensemble ML	LULC, demographics, traffic	~ 0.70	~ 3.5
Yelixiati et al.^32^	Block-scale analysis	Statistical models	BCR thresholds, patch metrics	0.65	3.1

*Note: RMSE values in most comparative studies reflect prediction of LST anomalies or urban-rural temperature differences rather than absolute LST*,* explaining the substantially lower errors compared to the present analysis.*

**Fig. 9:**
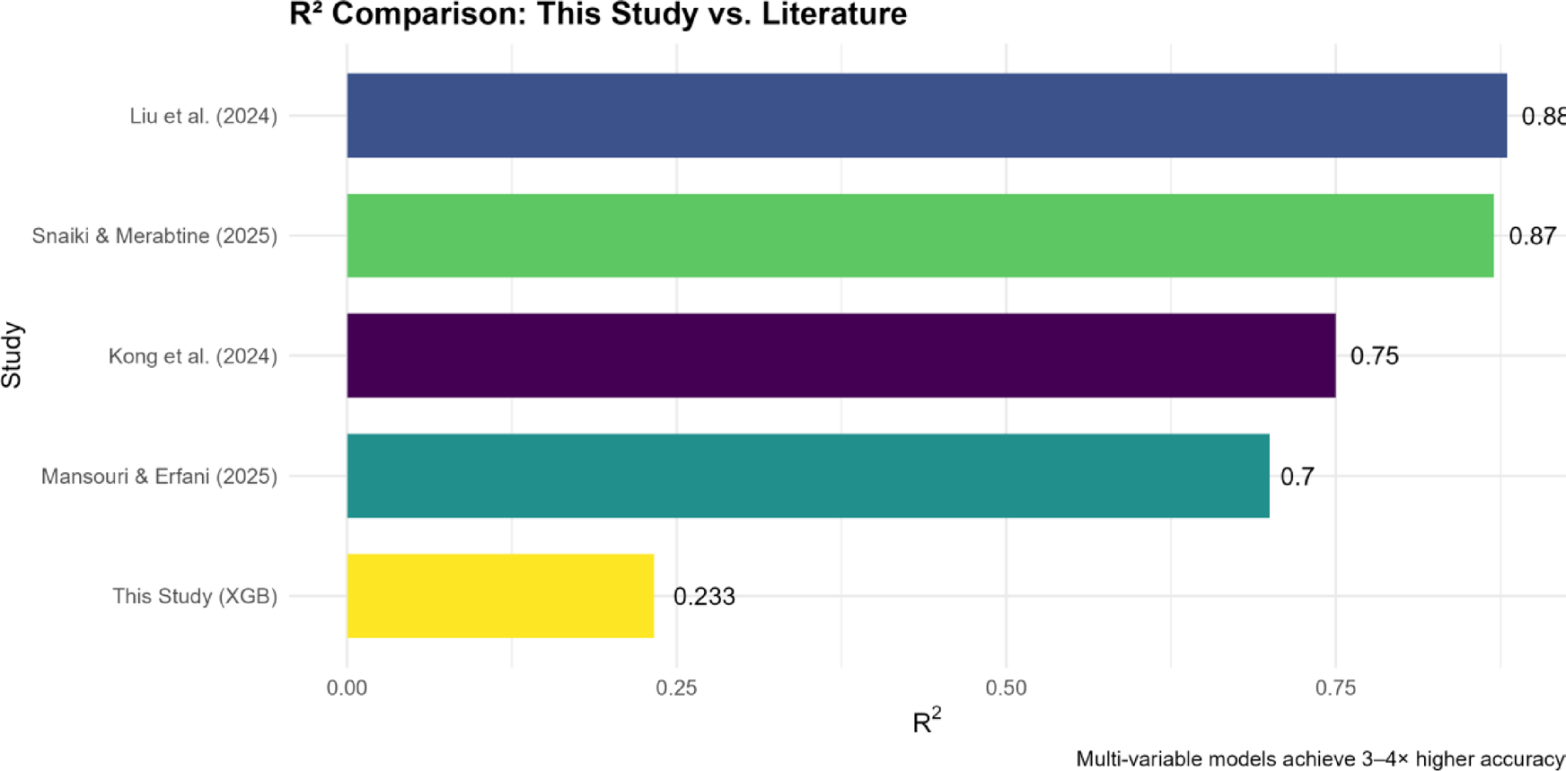
Comparison of R² values between the XGB model in this study and high-accuracy ML models from the literature. Multi-variable models achieve 3–4× higher accuracy, highlighting the impact of predictor diversity.

Figure 9, a bar chart comparing R² values, visually emphasizes that models incorporating meteorological variables, 3D urban form, or land use configuration achieve 3–4 times greater explanatory power than vegetation-only approaches.

Comparing this study with earlier works highlights the importance of integrating diverse predictors for accurate UHI modeling. Liu^31^, achieved R² = 0.88 using hybrid ML approaches that included landscape composition and configuration metrics, while Kong et al.^29^, obtained R² ≈ 0.75 across 216 global cities using deep-learning models that combined building height, albedo, and greenery information. In contrast, vegetation-only or two-dimensional morphology studies—such as Masoudi and Tan^33^ and Yelixiati et al.^32^,—typically report R² values below 0.30, comparable to the present analysis. This study contributes by offering a replicable open-data baseline specifically for subtropical high-density Asian contexts, where advanced 3D data are frequently unavailable and vegetation cooling can be constrained by local conditions.

These differences emphasize that vegetation indices alone cannot fully explain LST variability in dense urban environments. Multi-source data integration—including microclimate, 3D morphology, and anthropogenic heat—consistently enhances prediction accuracy^14,28^. The modest performance here is consistent with studies relying on limited 2D morphology metrics; incorporating a wider range of indicators (e.g., those used in analyses of seasonal/diurnal variations or plain/plateau contrasts) has yielded substantially higher R² values.

In addition, the results here are particularly consistent with studies from humid subtropical cities, where high humidity limits cooling efficiency and weakens NDVI–LST correlations^13,18^. While the achieved R² = 0.233 is modest compared with multi-variable/3D/multi-temporal studies (R² frequently > 0.7), it is consistent with the performance of vegetation + limited 2D morphology models in humid subtropical Asian contexts and serves as diagnostic evidence of the explanatory limits under open-data constraints common in many subtropical developing and middle-income cities. The principal contribution of this work is therefore to provide a replicable, fully open-data baseline analysis that quantifies the explanatory ceiling when advanced datasets are unavailable — a common situation in resource-constrained settings. This suggests that UHI modeling frameworks must be region-specific, accounting for local climatic constraints and morphological diversity.

### Policy and planning implications

Despite the moderate predictive performance of the models, the XGB results (Fig. 4b) offer indicative insights that may help identify priority areas for urban heat mitigation. High-LST zones with BCR > 0.5 and VegFrac < 0.3—mainly central commercial districts—should be prioritized for greening interventions. Partial dependence plots derived from the XGB model suggest that — holding other variables at their observed values — an increase of 10% points in VegFrac (e.g., from 0.30 to 0.40 in currently low-vegetation zones) is associated with an average marginal LST reduction of ≈ 1.0–1.5 °C during peak summer daytime conditions. This is a model-derived statistical association (not a causal estimate), and its magnitude is subject to considerable uncertainty given the modest overall R² (0.233), exclusion of important confounders (e.g., albedo, wind, anthropogenic heat), and the specific meteorological conditions of the single Landsat scene. Actual cooling from implemented greening interventions will depend on vegetation type, soil moisture status, surrounding morphology, and local ventilation. Therefore, the 1–1.5 °C range should be viewed as indicative guidance for prioritization rather than a precise or transferable prediction of intervention outcomes.

Moreover, the weak influence of BCR on LST suggests that promoting horizontal openness and ventilation corridors may be at least as important as reducing building density alone. Strategies such as preserving wind corridors, installing reflective “cool” roofs, and expanding urban vegetation may help mitigate UHI effects while supporting broader sustainability targets^14,35^. As a pragmatic open-data baseline, this study provides a low-cost starting point for identifying potential heat mitigation opportunities in resource-constrained subtropical cities like Osaka. These findings may offer supplementary insights for climate-resilient urban planning, contributing indirectly to Sustainable Development Goals (SDGs) 11 and 13.

## Limitations

This study has several limitations. First, the analysis used a single Landsat 9 scene (August 27, 2024), which restricts temporal representativeness. Multi-temporal and seasonal imagery would better capture vegetation phenology and improve understanding of seasonal cooling patterns^40,43^.

Second, the assumption of uniform building height (10 m) for BADR estimation likely introduces bias, as Osaka’s built environment varies considerably from low-rise residential to high-rise commercial structures^15,16^. More critically, restricting morphology to only BCR and BADR—while pragmatic given data constraints—omits key vertical and configurational dimensions (e.g., mean building height, floor area ratio, sky view factor, frontal area index, road density, landscape shape indices) commonly used in contemporary UHI research. This likely contributes substantially to the weak correlations and low overall model performance (highest R² = 0.233; RMSE = 29.598 °C), as these additional metrics have been shown to capture ventilation, shading, and pattern effects more effectively. Incorporating LiDAR-derived digital surface models would enhance 3D accuracy and better reflect the role of vertical morphology.

The uniform height assumption renders BADR statistically redundant with BCR, severely limiting its physical interpretability and conceptual justification in this context. Because BADR is directly computed as BCR multiplied by a constant height (10 m), the two variables are perfectly collinear (*r* = 1.000), offering no independent explanatory power. This dependency was anticipated and tested deliberately; the results confirm that basic planimetric metrics alone, without realistic vertical structure information, are insufficient for capturing meaningful morphology-related effects on LST in high-density subtropical settings. Future analyses should utilize open global DSM products such as ALOS AW3D30 (~ 30 m horizontal resolution, freely available from JAXA) to derive true mean building height, SVF, frontal area index, and other three-dimensional metrics, thereby overcoming the current limitations.

Third, the exclusion of meteorological factors such as wind speed, humidity, and solar radiation limits model completeness. Previous studies show that coupling morphological and meteorological parameters markedly improves UHI prediction in Tokyo and other Asian megacities^9,14^.

Fourth, the 100 m spatial resolution aggregates fine-scale variations within street canyons, potentially masking micro-climatic heterogeneity. Employing finer-resolution data (e.g., 30 m Sentinel-2 or UAV-based thermal imagery) could address this limitation^13,44^.

Finally, although advanced ML algorithms like XGB improved accuracy relative to linear models, the overall R² remained below 0.25. This underscores the complexity of urban thermal processes and highlights the potential of hybrid or physics-informed ML frameworks that incorporate surface-energy-balance constraints to ensure physically consistent predictions^23,25^. Future research should therefore prioritize multi-temporal satellite observations to resolve seasonal vegetation–temperature dynamics, LiDAR-derived or high-quality 3D building data for accurate vertical morphology representation, local-scale meteorological inputs, additional landscape and infrastructural metrics, and hybrid physics-informed machine learning architectures to enhance both predictive skill and physical interpretability.

## Conclusions and future research directions

The spatial relationships between LST and urban form–vegetation indicators in Osaka, Japan, were examined using high-resolution Landsat 9 imagery acquired on August 27, 2024, and OSM-derived building data. Weak positive correlations were observed between LST and vegetation metrics (Pearson’s *r* = 0.173 for NDVI_mean and *r* = 0.114 for VegFrac). This pattern is consistent with humid subtropical conditions during peak summer, where high atmospheric moisture can suppress latent heat flux and reduce vegetation cooling efficiency, further influenced by mixed land-cover composition within 100 m grid cells.

Building morphology indicators (BCR and BADR) exhibited near-zero correlation with LST (*r* ≈ 0.008), largely due to their restriction to 2D planimetric density and the uniform 10 m height assumption made in the absence of reliable 3D elevation data from OSM. This approach necessarily omits important vertical and configurational dimensions—such as building height variability, SVF, frontal area index, FAR, road density, and landscape pattern metrics (e.g., patch density, shape complexity)— that are known to influence radiative trapping, airflow, and thermal patterns in dense urban environments.

Nonlinear ML models substantially outperformed the MLR model, which explained only 4.5% of LST variance (R² = 0.045). Among the advanced approaches, XGB delivered the highest predictive accuracy on the independent test set, achieving R² = 0.233, RMSE = 29.598 °C, MAE = 20.782 °C, and MAPE = 106.992%. RF and ANN followed with R² = 0.205 and R² = 0.162, respectively. The elevated MAPE values arise from regression on absolute temperature (20–40 °C range) rather than anomalies and do not undermine model utility, with RMSE and R² serving as primary evaluative criteria. The modest explanatory power (highest R² = 0.233) aligns with expectations for analyses relying on vegetation indices and basic 2D morphology in humid subtropical settings. This study diagnostically demonstrates why planimetric density metrics add almost no value in this climatic and morphological setting, offering clear guidance on priority data needs (3D heights, landscape metrics, etc.) for future improvement.

Spatial LST predictions from the XGB model accurately identified thermal hotspots in central commercial districts characterized by high building coverage and minimal vegetation, while cooler signatures aligned with suburban parks and river-adjacent zones. Feature importance analysis within XGB revealed NDVI_mean as the dominant predictor (55.3% relative contribution), followed by VegFrac (44.7%), underscoring vegetation’s central—albeit constrained—role in modulating surface energy balance at the analyzed scale. Residual diagnostics confirmed XGB’s superior stability.

Although explanatory power remained modest, these results exceed linear benchmarks and align with findings from vegetation-focused UHI studies in compact Asian cities that rely on limited morphology metrics. The limited variance captured highlights the imperative for multi-variable integration in future modeling efforts. Incorporation of 3D urban morphology (e.g., mean building height, SVF, FAR), road density, landscape pattern indices, surface albedo, anthropogenic heat emissions, and meteorological forcings—approaches that have consistently yielded R² > 0.7 in broader frameworks—represents a critical pathway forward.

From an urban planning perspective, the analysis offers indicative support for considering vegetation enhancement in high-LST central zones. Partial dependence analysis from the XGB model suggests an indicative marginal LST reduction of approximately 1–1.5 °C associated with a 10% point increase in VegFrac (holding other variables constant). This is a model-derived association under the specific conditions of this single scene (peak summer, humid subtropical), not a causal or universally transferable estimate. Actual cooling outcomes will depend on vegetation type, soil moisture, surrounding morphology, and local ventilation. Given the weak linkage with 2D building density, strategies focusing on ventilation corridors, cool surface materials, green-blue infrastructure, and optimized 3D configurations may prove more effective than density reduction alone. These considerations may provide supplementary insights for climate-resilient planning and contribute indirectly to Sustainable Development Goals (SDGs) 11 and 13.

Building on the baseline established in this study using freely available data sources, the following concrete steps are recommended to advance UHI modeling accuracy and applicability in subtropical high-density cities:

- Integrate multi-temporal and seasonal satellite observations, including Landsat 9 time series combined with higher-frequency Sentinel-2 data (10–20 m resolution), to account for vegetation phenology, diurnal variations, and seasonal differences in LST–vegetation relationships.
- Incorporate open-access 3D elevation datasets such as the ALOS AW3D30 global DSM (freely available from JAXA at ~ 30 m horizontal resolution with ~ 5 m vertical RMSE), which provides sufficient quality for deriving mean building height, sky view factor (SVF), frontal area index, and other vertical morphology metrics in Osaka, overcoming the current uniform height assumption.
- Expand the predictor set to include additional open-data variables such as surface albedo (estimated from Landsat reflectance bands using established narrowband-to-broadband conversion formulas), distance to major water bodies (e.g., rivers and Osaka Bay for breeze effects), and anthropogenic heat flux proxies (from population density or energy consumption statistics).
- Adopt finer spatial resolution (e.g., 10–30 m) and explore hybrid modeling approaches that combine empirical machine learning with physics-informed constraints based on surface energy balance equations to improve physical interpretability and predictive skill.

These enhancements are expected to improve explanatory power and provide more robust insights for climate-resilient urban planning in data-constrained subtropical environments.

## Electronic Supplementary Material

Below is the link to the electronic supplementary material.

Supplementary Material 1

## Data Availability

The datasets generated and/or analysed during the current study are not publicly available due [Restrictions on Research Funding Supporting Institutions] but part of the raw datasets are available from the corresponding author on reasonable request.

## Abbreviations

ANN: Artificial Neural Network
BADR: Building Area Density Ratio
BCR: Building Coverage Ratio
CV: Cross-Validation
LST: Land Surface Temperature
MAE: Mean Absolute Error
MAPE: Mean Absolute Percentage Error
ML: Machine Learning
MLR: Multiple Linear Regression
NDVI: Normalized Difference Vegetation Index
OSM: OpenStreetMap
RF: Random Forest
RMSE: Root Mean Square Error
R²: Coefficient of Determination
SD: Standard Deviation
SDGs: Sustainable Development Goals
UHI: Urban Heat Island
VegFrac: Vegetation Fraction
VIF: Variance Inflation Factor
XGB: Extreme Gradient Boosting
DN: Digital number from thermal band
GAIN: Radiometric rescaling gain (0.00341802)
OFFSET: Radiometric rescaling offset (149.0)
$\:{\rho\:}_{NIR}$: Near-infrared band reflectance (Band 5)
$\:{\rho\:}_{Red}$: Red band reflectance (Band 4)
$\:{n}_{veg}$: Number of pixels with NDVI > 0.2
$\:{n}_{total}$: Total number of valid pixels in grid cell
$\:{A}_{building}$: Total building footprint area (m²)
$\:{A}_{cell}$: Grid cell area (100 m × 100 m)
$\:{V}_{building}$: Total building volume (m³)
$\:h$: Assumed building height (10 m)
$\:r$: Pearson correlation coefficient
$$\:{x}_{i},{y}_{i}$$: Paired observations
$$\:\stackrel{\prime }{x},\stackrel{\prime }{y}$$: Sample means
$\:\rho\:$: Spearman rank correlation coefficient
$$\:{d}_{i}$$: Rank difference
$\:n$: Number of observations/samples
$\:LS{T}_{i}$: Predicted land surface temperature
$$\:{\beta\:}_{0}$$: Intercept
$$\:{\beta\:}_{j}$$ (j = 1,…,4): Regression coefficients
$$\:{X}_{ij}$$: Predictor variables
$$\:{\epsilon}_{i}$$: Error term
$$\:{\widehat{y}}_{i}$$: Predicted value
$\:T$: Total number of trees
$\:{\widehat{y}}_{i}^{\left(t\right)}$: Prediction from tree t
$\:K$: Number of trees
$\:{f}_{k}$: Individual regression tree
$\:\mathcal{F}$: Space of all trees
$\:\stackrel{\prime }{y}$: Mean observed LST value
$$\:{x}_{j}$$: Input variables
$\:M$: Number of hidden neurons
$\:\sigma\:$: Sigmoid activation function
$$\:{w}_{kj},{v}_{j}$$: Weights
$$\:{b}_{k},c$$: Bias terms
$\:S{S}_{res}$: Residual sum of squares
$\:S{S}_{tot}$: Total sum of squares
BFGS: Broyden–Fletcher–Goldfarb–Shanno optimization method
