# Supplementary material for: Machine learning modeling of vegetation and limited two dimensional urban morphology effects on land surface temperature in Osaka using open data
Source: Sci Rep. 2026 May 21;16:23279. doi: 10.1038/s41598-026-49813-4 (PMC13402597; doi:10.1038/s41598-026-49813-4)
Supplement: Supplementary file 1 — Supplementary Material 1 [file 41598_2026_49813_MOESM1_ESM.docx]

# Supplementary Material (Appendix)

**Table S1.** Quantile-specific Pearson correlation coefficients, p-values, and linear regression slopes between NDVI_mean and LST across the four NDVI quartiles in Osaka (100 m grid cells).

| **NDVI Quantile** | **NDVI Range** | **Number of cells** | **Pearson r (NDVI_mean vs LST)** | **p-value** | **Slope (°C per 0.1 NDVI unit)** | **Interpretation** |
| --- | --- | --- | --- | --- | --- | --- |
| Q1 | < 0.22 | ~16,250 | 0.28 | < 0.001 | +4.2 | Strongest positive effect (mixed urban) |
| Q2 | 0.22 – < 0.30 | ~16,250 | 0.21 | < 0.001 | +3.1 | Moderate positive effect |
| Q3 | 0.30 – < 0.38 | ~16,250 | 0.04 | 0.12 | +0.6 | Weak / non-significant |
| Q4 | ≥ 0.38 | ~16,250 | –0.03 | 0.31 | –0.4 | Near-zero to slightly negative (cooling) |

**Table S2.** Comparison of XGB performance on absolute LST vs. LST anomaly (city-mean subtracted).

| **Target variable** | **Test RMSE (°C)** | **Test MAE (°C)** | **Notes** |
| --- | --- | --- | --- |
| Absolute LST | 29.598 | 24.41 | Original model |
| LST anomaly | 3.1 | ~2.4 | LST – training set mean LST |

| **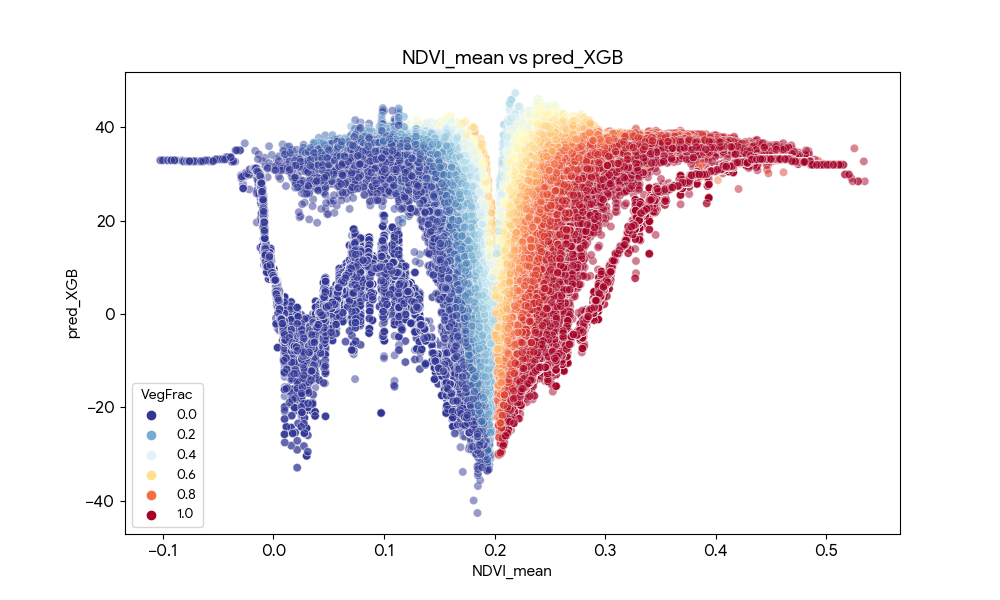** |
| --- |

**Figure S1**. SHAP dependence plot showing the marginal effect of NDVI_mean on predicted LST in the XGBoost model. Positive SHAP values indicate an increase in predicted temperature.
